# Changes in inflammatory cytokines, antioxidants and liver stiffness after chelation therapy in individuals with chronic lead poisoning

**DOI:** 10.1186/s12876-020-01386-w

**Published:** 2020-08-08

**Authors:** Tongluk Teerasarntipan, Roongruedee Chaiteerakij, Piyapan Prueksapanich, Duangporn Werawatganon

**Affiliations:** 1 Division of Gastroenterology, Department of Medicine, Faculty of Medicine, Chulalongkorn University and King Chulalongkorn Memorial Hospital, Bangkok, 10330 Thailand; 2 Center of Excellence for Innovation and Endoscopy in Gastrointestinal Oncology, Division of Gastroenterology, Department of Medicine, Faculty of Medicine, Chulalongkorn University, Bangkok, 10330 Thailand; 3 Alternative and Complementary Medicine for Gastrointestinal and Liver Diseases Research Unit, Department of Physiology, Faculty of Medicine, Chulalongkorn University, Bangkok, 10330 Thailand

**Keywords:** Chelation therapy, Chronic Lead poisoning, Hepatic fibrosis, Hepatic Steatosis, Oxidative stress, Hepatotoxicity

## Abstract

**Background:**

Chronic exposure to lead causes lead to accumulate mainly in the liver. In vivo studies have shown that lead toxicity is related to alterations in the inflammatory response. We aimed to evaluate the association between lead poisoning and liver fibrosis as well as the change in the degree of liver fibrosis, levels of inflammatory mediators and glutathione (GSH) after chelation therapy.

**Methods:**

Workers from a battery factory who were exposed to lead for > 12 months and had a blood lead level (BLL) > 70 μg/dL were enrolled (*n* = 86) in the study. Participants underwent chelation therapy with intravenous CaNa_2_EDTA for 2 days followed by treatment with oral D-penicillamine for 90 days. The primary outcome was the change in the degree of liver fibrosis, which was presented as liver stiffness (LS) measured by FibroScan®. Secondary outcomes were the changes in the levels of serum GSH and inflammatory mediators such as tumor necrosis factor-alpha (TNF-α), interleukin-1β (IL-1β), and interleukin-6 (IL-6) after chelation therapy.

**Results:**

Among the 86 participants, there was a positive correlation between the duration of lead exposure and LS (r = 0.249, *p* = 0.021). To avoid the confounding effect of obesity-related steatosis, only 70 individuals who had controlled attenuation parameters < 296 dB/m, BMI < 25 kg/m^2^ and normal waist circumference were included in the interventional analysis. After chelation, the mean LS significantly decreased from 5.4 ± 0.9 to 4.8 ± 1.4 kPa (*p* = 0.001). Similarly, all of the inflammatory cytokines studied significantly decreased after chelation (*p* < 0.001); TNF-α decreased from 371.6 ± 211.3 to 215.8 ± 142.7; the levels of IL-1β decreased from 29.8 ± 1.7 to 25.9 ± 4.3; and the levels of IL-6 decreased from 46.8 ± 10.2 to 35.0 ± 11.9. On the other hand, the mean GSH level increased significantly from 3.3 ± 3.3 to 13.1 ± 3.7 (*p* < 0.001) after chelation therapy.

**Conclusion:**

The duration of lead exposure was significantly correlated with the degree of liver fibrosis. Chelation treatment was associated with increased levels of GSH and decreased levels of proinflammatory cytokines and could potentially reduce the degree of LS.

**Trial registration:**

This study was retrospectively registered and approved by the Thai Clinical Trial Registry (TCTR) on 2019-11-07. The TCTR identification number is TCTR20191108001.

## Background

Lead is a heavy metal that can be found in the environment at low concentration levels. However, in the industrialization era, lead is widely used in a variety of products, such as batteries, gasoline, and ceramics [1]. Workers involved in these manufacturing processes are therefore at higher risk of developing lead toxicity. In a recent article, the authors were concerned that the prevalence of lead poisoning was underestimated in low- and middle-income countries [2, 3]. Chronic lead poisoning, although uncommon, is associated with nonspecific symptoms such as constipation, anorexia and recurrent colicky abdominal pain with an insidious onset, and chronic lead poisoning affects multiple organ systems [4]. Hence, many cases are misdiagnosed or detected in late stages.

Most previously reported cases of lead-induced liver injury were associated with mild and self-limited hepatitis. However, limited studies have been conducted in humans that assess the effects of lead poisoning on the liver [5–7]. All previous studies focused on the abnormalities in liver function tests that represented only liver injury at one time point. Since the liver is one of the major reservoirs of lead accumulation, lead poisoning can cause chronic liver injury. Few lead intoxication animal studies have investigated the pathology of liver fibrosis and steatosis. In animal models, a reduction in antioxidants, particularly glutathione (GSH), was identified and was found to be the main mechanism underlying lead-induced hepatoxicity [7]. In addition, it was shown that animals with chronic lead exposure had elevated proinflammatory cytokines, such as interleukin (IL)-1β, IL-6, IL-8, IFN-γ and tumor necrosis factor (TNF)-α [7].

As a result, the primary aim of our study was to evaluate the effects of chronic lead toxicity on the liver, specifically in regard to fibrosis and steatosis. This study assessed the levels of hepatic fibrosis and steatosis as well as the levels of inflammatory cytokines and antioxidants before and after chelation therapy.

## Methods

### Study design

This study was performed during an initial survey of lead toxicity among individuals who had worked in a battery-manufacturing factory with standard occupational health policies. This study was conducted in two phases. The first phase involved a cross-sectional cohort of participants with severe chronic lead poisoning. We determined the associations between chronic lead poisoning and liver injury. The second phase was a prospective interventional cohort that evaluated whether chelation therapy could reduce lead-related liver injuries. Both studies were conducted from August 1, 2018 to February 1, 2019. In parallel with the study, the lead toxicity prevention program of the factory was re-evaluated and reinforced by occupational medicine specialists to prevent further lead exposure.

### Study cohorts

#### Phase I: initial cross-sectional cohort

This cohort study aimed to evaluate associations between severe chronic lead poisoning parameters and potential adverse effects on the liver. The inclusion criteria were as follows: (1) age ≥ 18 years; (2) occupational exposure to lead in the battery factory for ≥ 12 months; and (3) blood lead level (BLL) ≥ 70 μg/dl, which is the cut-off level for severe occupational chronic lead poisoning and should be treated with intravenous chelation therapy according to the US Occupational Safety and Health Administration (OSHA) [8]. We excluded participants who had chronic liver diseases such as viral hepatitis B or C (HBV or HCV) infection, autoimmune hepatitis, Wilson’s disease, hemochromatosis, or alcoholic liver disease (i.e., history of alcohol consumption ≥ 30 g/day in men or ≥ 20 g/day in women for at least 3 months within 1 year prior to enrollment) and nonalcoholic fatty liver disease (NAFLD). Participants who had risk factors for NAFLD, such as diabetes mellitus, serum triglycerides ≥ 200 mg/dL, and/or waist circumference (WC) ≥ 102 cm in men or ≥ 88 cm in women, were also excluded. Other exclusion criteria were use of possible hepatotoxic medication within 12 months prior to study enrollment, previous chelation therapy, and presence of signs or symptoms of acute lead poisoning such as colicky abdominal pain, hemolytic anemia, and polyneuropathy.

All workers from the battery factory were screened for BLL (*n* = 720). Participants who met the inclusion and exclusion criteria were enrolled in the initial cross-sectional cohort (*n* = 86).

#### Phase II: prospective interventional cohort

This interventional cohort aimed to evaluate the mechanisms underlying liver injury caused by chronic lead poisoning by comparing the change in liver stiffness (LS) and amount of hepatic fat based on the changes in the amount of GSH and proinflammatory cytokines. Since fatty liver is a well-known factor that contributes to liver fibrosis, we excluded 16 participants with severe fatty liver, which is defined as having controlled attenuation parameters (CAP) > 296 dB/m or high-risk features for metabolic syndrome such as BMI > 25 kg/m^2^ and WC > than the cut-off criteria for metabolic syndrome according to the National Cholesterol Education Program Adult Treatment Panel III (NCEP ATP III) (i.e., WC > 80 cm for women and WC > 90 cm for men) to eliminate the effects of confounding factors for obesity-related steatosis. The remaining 70 eligible participants were then enrolled in the prospective interventional cohort. The participants received 2 g of CaNa_2_EDTA intravenously for 2 days followed by 1 g/day oral D-penicillamine for 90 days. The treatment regimen was a modified regimen designed by our institute’s clinical toxicologist in accordance with the drug profiles and patients’ medical adherence. All participants were advised to avoid potential hepatotoxic medications and alcohol consumption. The primary outcome was the change in LS and steatosis after chelation therapy. The levels of GSH and inflammatory cytokines were prospectively evaluated on the last day of treatment. Secondary outcomes were the correlations among the change in BLL, liver steatosis, liver fibrosis, GSH and inflammatory markers between the pre- and postchelation therapy timepoints. The investigators and the participants were blinded to the results of the blood tests and FibroScan® (Echosens, Paris, France).

#### Data and specimen collection

Clinical information, complete blood count, liver function test, and serum samples were collected upon admission. Noninvasive liver assessments were performed by a certified single operator. FibroScan® (transient elastography (TE)) was used to assess liver stiffness (LS) and the degree of liver steatosis, which was presented as controlled attenuation parameters (CAPs).

**Fig. 1:**
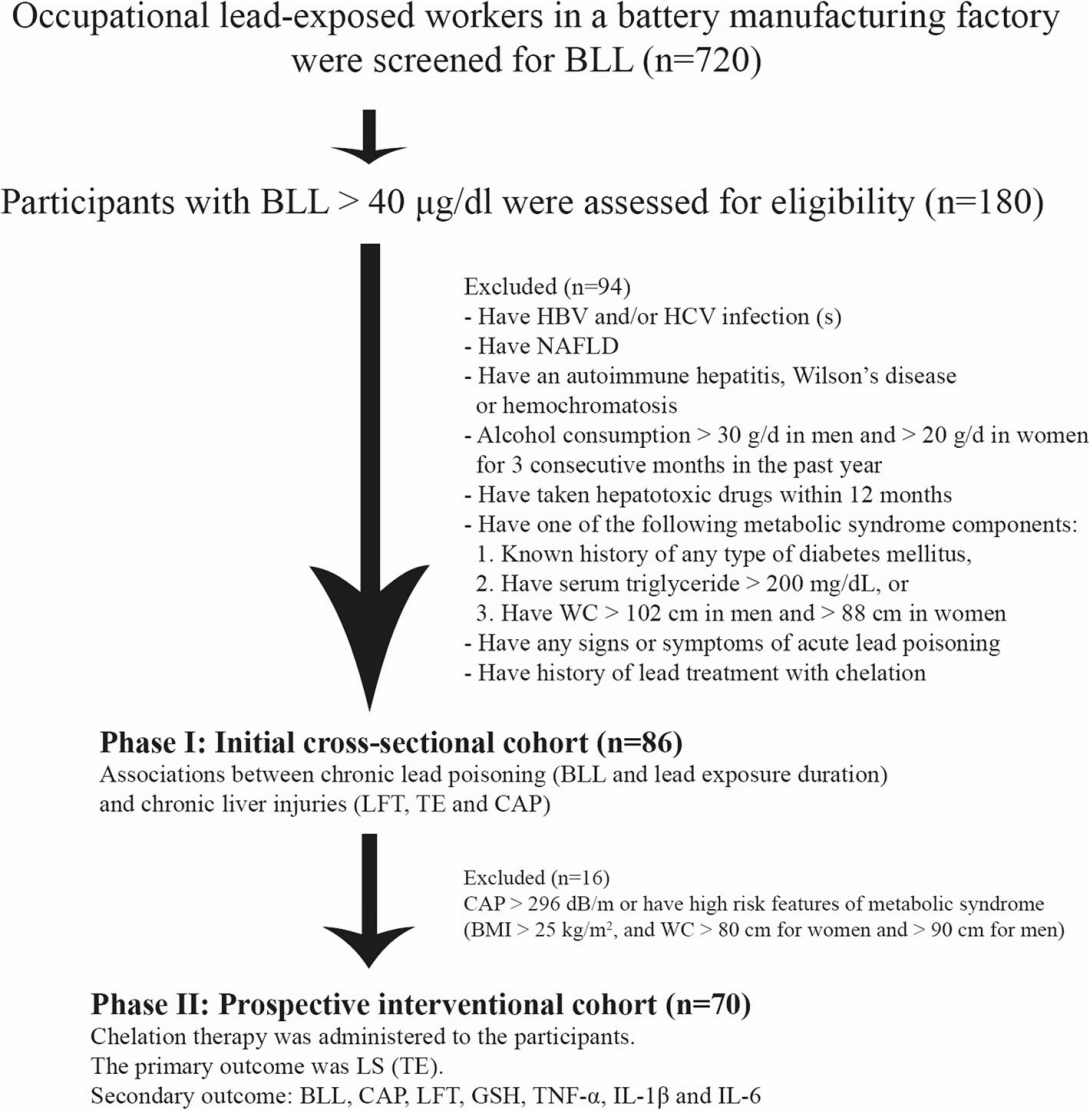
Study flow

Serum samples from pre- and postchelation were stored at − 80 °C until analysis. The levels of GSH, TNF-α, IL-1β, and IL-6 were measured using a solid-phase enzyme immunoassay technique using commercially available kits (R&D Systems, Inc., Minneapolis, MN, USA) according to the manufacturer’s protocol. The absorbance was read at 450 nm. The flow of the study is shown in Fig. 1.

### Statistical analysis

For testing differences between two dependent means, the estimated sample size was based on the results of a pilot study of 10 workers with chronic lead poisoning that was performed at our hospital; the mean LS was 5.2 + 0.7 kPa. The reference value of 5.5 + 3.8 kPa in the control group was derived from a survey among 782 healthy Thai volunteers [8]. The calculated minimum sample size was 58 cases for 80% power. Categorical data were compared using Fisher’s exact test. Continuous variables are described as the means and standard deviations. Potential relationships between lead-related parameters and the degree of hepatic fibrosis and steatosis were initially assessed using Pearson’s correlation analysis. Statistically significant parameters were subsequently included in the multivariate linear regression analysis. A dependent samples t-test was used to compare the results between pre- and posttreatment. Pearson’s correlation analysis was used to find the correlation between the percentage changes in BLL and the percentage changes in the levels of inflammatory biomarkers.

## Results

### Phase I: initial cross-sectional cohort

#### Baseline characteristics

BLL was screened in a total of 720 participants. A total of 180 participants had a BLL > 40 μg/dl. This level indicated that chronic lead toxicity was present and that chelation therapy was required (Fig. 1). Among the 180 participants, 86 participants met the inclusion and exclusion criteria and were enrolled in the study. Table 1 shows the baseline characteristics of the 86 participants. Most of the participants were males (*n* = 71, 85%), and the average age was 37.6 ± 7.3 years. The mean BLL was 81.4 ± 9.8 μg/dl. Twenty-six (30.2%) participants had hepatitis; they had SGOT levels above the ULN (> 35 mg/dl, *n* = 14) and/or SGPT above the ULN (> 40 mg/dl, *n* = 17).

**Table 1:** Baseline pretreatment laboratory profiles of 86 participants in the initial cross-sectional cohort

Parameters	Mean ± SD
Age (years)	37.6 ± 7.3
Waist (cm)	80.2 ± 12.0
BMI (kg/m^2^)	24.2 ± 4.9
Blood lead level (μg/dL)	81.4 ± 9.8
Hemoglobin (g/dL)	13.4 ± 1.6
White blood cell count (10^3^/μL)	8.0 ± 1.8
Platelets (10^12^/L)	285.6 ± 58.4
Creatinine (mg/dL)	0.9 ± 0.3
Total bilirubin (mg/dL)	0.8 ± 0.4
Direct bilirubin (mg/dL)	0.3 ± 0.1
Alkaline phosphatase (U/L)	68.8 ± 19.5
SGOT (U/L)	30.7 ± 28.9
SGPT (U/L)	33.3 ± 33.2
Liver stiffness (kPa)	5.4 ± 0.9
CAP (dB/m)	225.1 ± 49.3

*Abbreviations*: *SGOT* serum glutamate oxaloacetate transaminase, *SGPT* serum glutamate pyruvate aminotransferase, *CAP* controlled attenuation parameters

**Fig. 2:**
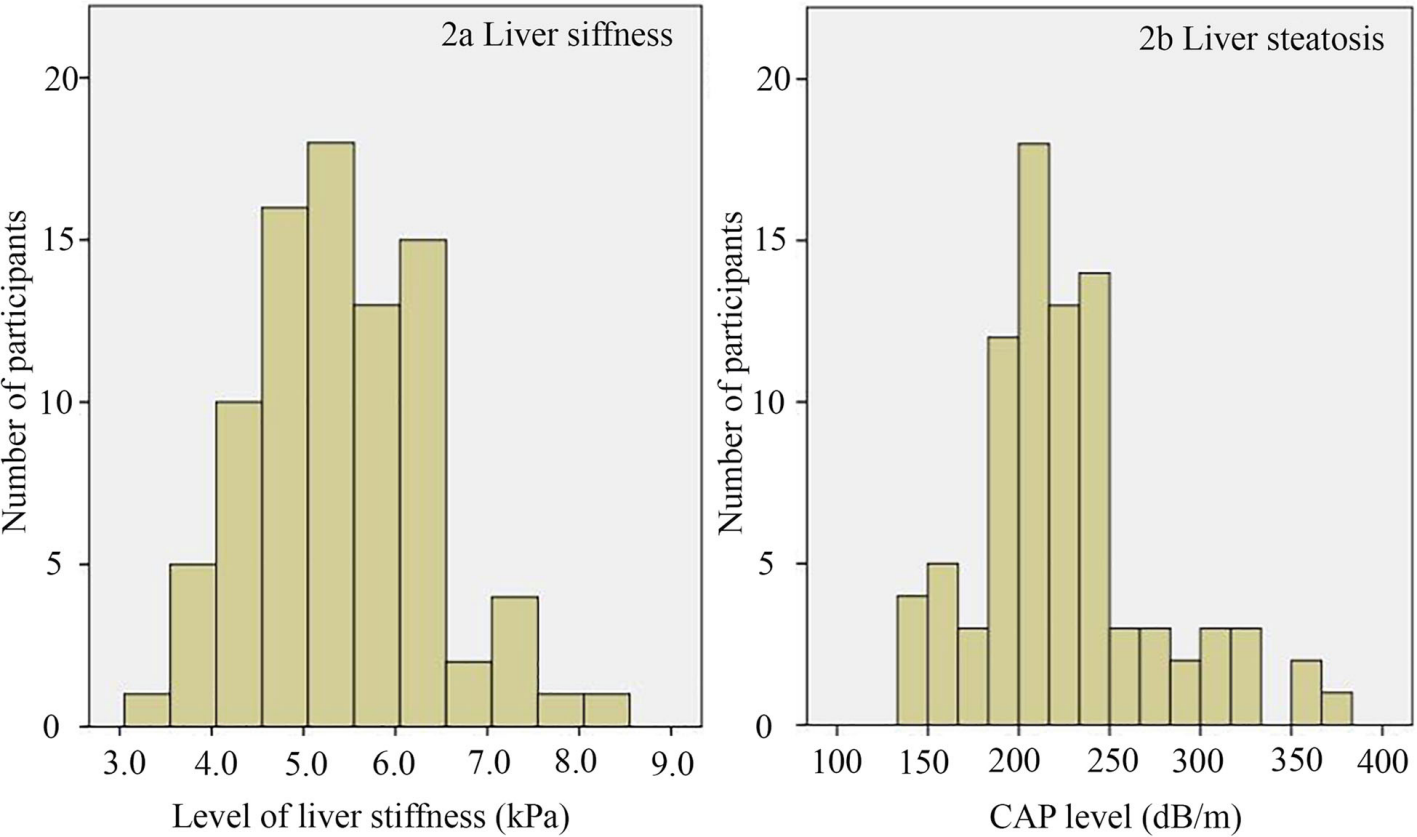
Distribution of the number of study participants from initial cohorts. **a** Proportion of study participants and their level of liver stiffness (LS). **b** Proportion of study participants and their level of steatosis (CAP)

The mean LS value of the 86 participants was 5.4 ± 0.9 kPa. Notably, 23 participants (26.7%) from this cohort had significant fibrosis (i.e., the LS value was > 6.1 kPa). The mean CAP was 225.1 ± 49.3 dB/m. Forty-four (51.2%) of the participants had CAP > 213 dB/m, indicating that there was significant liver steatosis. Among those with significant fibrosis, 42 participants (48.8%) had no steatosis (S0), while 30 (34.8%) and 15 (17.4%) participants had mild-moderate steatosis (S1–2) and severe steatosis (S3), respectively. The numbers of participants with LS and CAP are shown in Fig. 2.

### Factors associated with liver fibrosis and steatosis

In the univariate analysis, the duration of lead exposure, but not BLL, was significantly associated with the degree of hepatic fibrosis (Pearson’s r = 0.249, *p* = 0.021). Other factors associated with liver fibrosis included age, BMI, liver steatosis, SGPT, and ALP (Table 2). In the multivariate analysis, only the duration of lead exposure and the SGPT level remained independently associated with liver fibrosis, with Pearson’s r values of 0.229 and 0.317 (*p* = 0.026 and 0.002), respectively.

**Table 2:** Potential factors that may be associated with liver fibrosis and steatosis in the initial cross-sectional cohort

Parameters	Factors associated with liver fibrosis				Factors associated with liver steatosis			
	Univariate analysis		Multivariate analysis		Univariate analysis		Multivariate analysis	
	Pearson Correlation	*P*-value	Pearson Correlation	*P*-value	Pearson Correlation	*P*-value	Pearson Correlation	*P*-value
Age	0.215	0.047	0.195	0.060	- 0.042	0.703	–	–
Sex	- 0.155	0.153	–	–	-0.237	0.028	-0.161	0.062
BLL	-0.086	0.430	–	–	-0.075	0.493	–	–
Duration of lead exposure	0.249	0.021	0.229	0.026	0.020	0.854	–	–
Waist circumference	0.220	0.076	–	–	0.702	< 0.001	0.524	< 0.001
BMI	0.226	0.037	0.107	0.350	0.660	< 0.001	2.064	0.360
Liver steatosis	0.242	0.025	0.080	0.525	1	–	–	–
Liver fibrosis	1	–	–	–	0.242	0.025	2.155	0.656
SGOT	0.161	0.142	–	–	0.329	0.002	−0.531	0.273
SGPT	0.332	0.002	0.317	0.002	0.566	< 0.001	0.397	0.018
ALP	0.222	0.041	0.107	0.347	0.154	0.161	–	–

*Abbreviations*: *BLL* blood lead level, *BMI* body mass index, *SGOT* serum glutamate oxaloacetate transaminase, *SGPT* serum glutamate pyruvate aminotransferase, *ALP* alkaline phosphatase

The following variables were found to be significantly associated with the presence of liver steatosis in the univariate model: sex, WC, BMI, liver fibrosis, SGOT, and SGPT. WC and SGPT were independently associated with liver steatosis, with Pearson’s r values of 0.524 and 0.397 (*p* < 0.001 and 0.018), respectively. We did not detect any association between lead-related parameters and liver steatosis (Table 2).

### Phase II. Interventional prospective cohort

#### Effects of chelation therapy on liver fibrosis and steatosis

An association between liver fibrosis and steatosis was detected in our initial cross-sectional cohort (Pearson’s r = 0.242, *p* = 0.025). After excluding participants with severe fatty liver and those with high-risk features for metabolic syndrome as described previously**,** a total of 70 participants were enrolled in this study. Correlation analyses were repeated to confirm the independent effects between the degree of liver fibrosis and steatosis. We found that there was no significant association between the degree of fibrosis and steatosis. The Pearson’s r between the level of LS and CAP at the prechelation phase was - 0.039 (*p* = 0.75), and at the postchelation phase, it was 0.151 (*p* = 0.21). Pearson’s correlation analysis between the degree of postchelation LS reduction and the degree of postchelation CAP reduction was 0.160 (*p* = 0.19) (Supplementary Table 1).

After 3 months of chelation therapy, the mean BLL decreased from 81.8 + 9.9 to 56.6 + 16.8 μg/dL (30.8%). After treatment, the degree of LS decreased significantly from 5.33 + 0.9 to 4.8 + 1.4 kPa (*p* = 0.001). We did not find significant improvement in liver steatosis after chelation therapy (mean pre- and postchelation CAP levels were 208.6 + 31.7 and 207.0 + 45.0 dB/m, *p* = 0.738, respectively) (Table 3).

**Table 3:** Comparison of lead-related parameters between pre- and postchelation therapy

Parameters	Prechelation (mean + SD)	Postchelation (mean + SD)	Mean difference between post- and prechelation (95% confidence interval)	*p*-value
Blood lead level (μg/dL)	81.8 ± 9.9	56.6 ± 16.8	-25.2 + 13.8 (21.9–28.5)	< 0.001
Liver stiffness (kPa)	5.3 ± 0.9	4.8 ± 1.4	- 0.5 + 1.2 (0.2–0.8)	0.001
Steatosis (dB/m)	208.6 ± 31.7	207.0 ± 45.0	-1.6 + 41.0 (-8.1–11.4)	0.738
TNF-α (pg/mL)	371.6 ± 211.3	215.8 ± 142.7	-155.8 + 137.4 (120.9–190.7)	< 0.001
Interleukin-1β (pg/mL)	29.8 ± 1.7	25.9 ± 4.3	-3.8 + 3.7 (2.9–4.8)	< 0.001
Interleukin-6 (pg/mL)	46.8 ± 10.2	35.0 ± 11.9	-11.8 + 10.6 (9.1–14.5)	< 0.001
Glutathione (μg/mL)	3.3 ± 3.3	13.1 ± 3.7	9.8 + 3.7 (10.8–8.9)	< 0.001

*Abbreviation*: *TNF* tumor necrosis factor

#### Effects of chelation therapy on oxidative stress and inflammatory markers

The mean levels of the inflammatory biomarkers TNF-α, IL-1β and IL-6 were significantly reduced after chelation therapy by 41.93% (371.6 + 211.3 to 215.8 + 142.7 pg/mL), 13.09% (29.8 + 1.7 to 25.9 + 4.3 pg/mL), and 25.21% (46.8 + 10.2 to 35.0 + 11.9 pg/mL), respectively. On the other hand, the mean GSH level significantly increased after chelation therapy from 3.3 + 3.3 to 13.1 + 3.7 μg/mL (297.0%) (Table 3). However, the correlation between the degree of change in BLL and the reductions in TNF-α and IL-6 levels was not significant. The increase in the level of GSH was also not significant (Table 4).

**Table 4:** Degree of correlation between reduced BLL, LS, and CAP for each inflammatory marker studied

Inflammatory markers affected by chelation treatment	Reduced BLL		Reduced LS		Reduced CAP	
	Pearson’s Correlation	*p*-value	Pearson’s Correlation	*p*-value	Pearson’s Correlation	*p*-value
TNF-α	0.212	0.919	-0.014	0.909	-0.237	0.048
Interleukin-1β	0.034	0.778	0.045	0.714	0.008	0.945
Interleukin-6	0.118	0.332	0.020	0.872	0.055	0.652
Glutathione	-0.100	0.410	-0.030	0.802	-0.079	0.517

*Abbreviations*: *BLL* blood lead level, *LS* liver stiffness, *CAP* controlled attenuation parameters, *TNF* tumor necrosis factor

## Discussion

Each day, approximately 0.1–2 mg of lead enters the human body through ingestion (75%), inhalation and skin contact (25%). Once lead is absorbed and enters the bloodstream, it is distributed and deposited in various types of soft tissue in the human body. Lead accumulates in the bone, followed by the liver, kidney, neurons, and spleen [9].

In the blood, 95% of lead binds to erythrocytes and has a mean half-life of 35 days. There are various ranges of normal BLL depending on an individual’s age and environmental exposure to lead. BLLs of 25–40 μg/dl in adults and 5–10 μg/dL in children are considered to be normal reference levels in the nonlead-exposure population, while a BLL of 40–60 μg/dl is an acceptable normal value among occupational lead-exposure workers [10]. The diagnosis of chronic lead poisoning is based on BLL regardless of the presence of signs or symptoms.

To date, there are few studies about hepatotoxicity from lead poisoning. Most of these studies were case reports of participants with acute lead poisoning symptoms and abnormal liver chemistry tests; the ranges of the liver enzymes for SGOT and SGPT were 63–66 mg/dL and 75–256 mg/dL, respectively [5–7, 11–16]. No liver failure was reported. There were only four analytical studies that focused on hepatotoxicity among occupational lead-exposed workers [4–6, 17]. Every study found that there was a mild elevation of liver enzymes. Two of the studies showed significant differences in the levels of liver enzymes between occupational lead-exposed workers and healthy control participants [6, 7]. However, in the other two studies, there were no differences in the levels of liver enzymes between the people who were exposed to lead and those in the control group [5, 17]. In our study, the majority of participants had normal levels of liver enzymes. However, approximately 20% of the participants had elevated SGOT and/or SGPT levels without any known causes. Bilirubin and ALP levels were also normal. These findings were in concordance with previous reports [4–6, 17]. Lead accumulates in the liver among workers who are constantly exposed to lead. Therefore, we recommend that further investigations exploring chronic toxicity from chronic lead poisoning be conducted.

Our study found that GSH levels were markedly elevated and that BLL decreased after chelation therapy. This might imply that lead depleted antioxidants, which was consistent with findings from animal studies [10, 18]. Lead-induced oxidative stress was the main mechanism of lead poisoning according to the animal models; there was a decrease in GSH reserve and an increase in reactive oxygen species (ROS). Lead inactivates GSH by binding to sulfhydryl groups and inhibits GSH synthesis [9, 18]. In addition, lead destabilizes the cell membrane by inducing lipid peroxidation, changes the membrane’s biophysical properties and causes cell damage [18]. Our study was the first human study that supported findings from animal studies demonstrating that GSH depletion contributed to liver injury in individuals with chronic lead poisoning.

The results from our study supported the systemic inflammation theory. We showed that after chelation therapy, the BLL and the levels of proinflammatory cytokines such as TNF-α, IL-1β and IL-6 were reduced. Lead exposure could enhance the production of various proinflammatory cytokines, such as IL-1β, IL-6, IL-8, IFN-γ and TNF-α. Overall, lead causes tissue damage by inducing inflammation and inhibits anti-inflammatory mechanisms [19–23].

The liver is one of the major organs that accumulates lead. We hypothesized that sustained lead exposure contributed to chronic inflammation, which predisposed individuals to hepatic fibrosis. Histopathological findings from animal experiments [23, 24] and two human case reports of acute lead poisoning with unexplained hepatitis demonstrated extensive microvesicular and macrovesicular steatosis, portal and intralobular lymphocytic infiltrate, disrupted liver parenchymal architecture and pericellular fibrosis [12, 24]. Our study used LS as a noninvasive parameter that represented the degree of liver fibrosis. Although the mean LS in our study was within the normal range, 26.7% of the participants had LS values above the significant fibrosis cut-off level. Notably, 82.6% of the participants with significant fibrosis had nonsevere steatosis; thus, significant liver fibrosis might be a consequence of lead poisoning. Our study found that the duration of lead exposure was the major factor associated with the development of liver fibrosis. This finding supported our hypothesis that liver injury occurred as a result of chronic lead poisoning.

After chelation therapy, we found that the degree of LS and levels of inflammatory cytokines were significantly reduced and that there was an increase in GSH. These findings suggested that liver fibrosis was associated with lead poisoning. Although the change in the degree of LS was not significantly altered in proportion to a change in any single biomarker, we postulated that each cytokine exerted small effects in concert, rather than a single cytokine resulting in hepatic fibrosis.

Evidently, chronic liver inflammation from chronic lead poisoning not only leads to hepatic fibrosis but also induces various pathways that contribute to the development of hepatic steatosis. Animal studies have revealed that lead-intoxicated rats had altered gene expression of hepatic enzymes involved in cholesterol and triglyceride homeostasis^7,25–27^. Few studies have found significant hypertriglyceridemia and hypercholesterolemia among lead-exposed workers^28, 29^ with scant histological reports of macrovesicular steatosis [12]. The mean CAP in our study was 225.1 + 49.3 dB/m, which was considered to indicate mild steatosis (S1). Notably, 54.7% of our participants had significant steatosis. However, we did not find a significant correlation between the degree of steatosis and lead-associated parameters, such as the duration of exposure and BLL. Obesity might overcome the effects of chronic lead poisoning. We observed a strong significant correlation between the degree of steatosis and BMI as well as WC.

Regarding the change in the degree of liver steatosis, we did not find a significant reduction in the level of CAP after treatment. In terms of inflammatory marker analysis, only TNF-α levels changed and were significantly negatively correlated with CAP changes. It should be noted that the mean prechelation CAP level was rather low and within the normal reference range. It is possible that the sample size was too small to allow for the detection of a change in CAP after therapy. Thus, we cannot confidently conclude that there was no relationship between lead toxicity and steatosis.

Our study has some limitations. First, the lack of a control group in our study might compromise the strength of the conclusion of the efficacy of chelation therapy in regard to changes in the degree of liver fibrosis, levels of inflammatory mediators and GSH. Regarding liver fibrosis and steatosis detection, we did not use the gold standard of histopathology to detect liver fibrosis and steatosis. Due to ethical and safety concerns, we opted to use a noninvasive technique, FibroScan®, which has been validated by other investigators. FibroScan® has shown good accuracy in evaluating the degree of fibrosis and can even replace liver biopsy^30^. Another limitation was that the chelation regimen might be insufficient because the mean posttreatment BLL was still above the normal level, and the follow-up time might have been too short to detect any change after treatment.

## Conclusion

Continuous exposure to lead has an adverse effect on the liver. The duration of lead exposure was significantly correlated with the degree of fibrosis. Lead might deplete antioxidants and increase the systemic inflammatory response. Treatment with chelation was associated with increased levels of GSH and decreased levels of proinflammatory cytokines such as TNF-α, IL-1β and IL-6. Hence, chelation therapy could potentially reduce the degree of LS.

## Supplementary information

**Additional file 1: Supplementary Table 1.** Correlation analyses between LS and CAP in the prospective intervention cohort

## Data Availability

The datasets used and/or analyzed during the current study are available from the corresponding author on reasonable request.

## Abbreviations

GSH: Glutathione
LS: Liver stiffness
TNF: Tumor necrosis factor
IL: Interleukin
BLL: Blood lead level
US: United States
OSHA: Occupational Safety and Health Administration
HBV: Hepatitis B virus
HCV: Hepatitis C virus
NAFLD: Nonalcoholic fatty liver disease
WC: Waist circumference
CAP: Controlled attenuation parameters
NCEP ATP: National Cholesterol Education Program Adult Treatment Panel
TE: Transient elastography
ULN: Upper limit normal
SGPT: Serum glutamate-pyruvate transaminase
ALP: Alkaline phosphatase
BMI: Body mass index
SGOT: Serum glutamic-oxaloacetic transaminase
