# Correction to: Changes in inflammatory cytokines, antioxidants and liver stiffness after chelation therapy in individuals with chronic lead poisoning

**DOI:** 10.1186/s12876-021-01634-7

**Published:** 2021-03-05

**Authors:** Tongluk Teerasarntipan, Roongruedee Chaiteerakij, Piyapan Prueksapanich, Duangporn Werawatganon

**Affiliations:** 1 Division of Gastroenterology, Department of Medicine, Faculty of Medicine, Chulalongkorn University and King Chulalongkorn Memorial Hospital, Bangkok, 10330 Thailand; 2 Center of Excellence for Innovation and Endoscopy in Gastrointestinal Oncology, Division of Gastroenterology, Department of Medicine, Faculty of Medicine, Chulalongkorn University, Bangkok, 10330 Thailand; 3 Alternative and Complementary Medicine for Gastrointestinal and Liver Diseases Research Unit, Department of Physiology, Faculty of Medicine, Chulalongkorn University, Bangkok, 10330 Thailand

## Correction to: BMC Gastroenterology (2020) 20:263 10.1186/s12876-020-01386-w

Following publication of the original article [1], the authors identified an error in corresponding author marked in the articles. The correct corresponding author is as follows:

Tongluk Teerasarntipan^1^, Roongruedee Chaiteerakij^1,2^, Piyapan Prueksapanich^1,*^, Duangporn Werawatganon^3^
