# Supplementary material for: Changes in inflammatory cytokines, antioxidants and liver stiffness after chelation therapy in individuals with chronic lead poisoning
Source: BMC Gastroenterol. 2020 Aug 8;20:263. doi: 10.1186/s12876-020-01386-w (PMC7414709; doi:10.1186/s12876-020-01386-w)
Supplement: Supplementary file 1 — Additional file 1: Supplementary Table 1. Correlation analyses between LS and CAP in the prospective intervention cohort [file 12876_2020_1386_MOESM1_ESM.docx]

Supplementary table 1 Correlation analyses between LS and CAP in the prospective intervention cohort

| Parameters | Pre-chelation | | Post-chelation | | Difference between pre-and post-chelation | |
| --- | --- | --- | --- | --- | --- | --- |
|  | Pearson’s correlation | p-value | Pearson’s correlation | p-value | Pearson’s correlation | p-value |
| LS and CAP | -0.039 | 0.75 | 0.151 | 0.214 | 0.160 | 0.187 |

LS; liver stiffness, CAP; controlled attenuation parameters
